# Evaluation of microbial contamination on cuff syringe, cuff pressure gauge, and their surroundings in the operating room

**DOI:** 10.1186/s40981-021-00486-0

**Published:** 2021-12-07

**Authors:** Rieko Oishi, Kiwamu Nakamura, Yoko Yahagi, Kazutaka Ohashi, Yukiko Takano, Rie Harada, Shinju Obara, Satoki Inoue, Keiji Kanemitsu, Masahiro Murakawa

**Affiliations:** 1 Department of Anesthesiology, Fukushima Medical University, 1 Hikarigaoka, Fukushima-city, Fukushima Japan; 2 Department of Infection Control, Fukushima Medical University, 1 Hikarigaoka, Fukushima-city, Fukushima Japan; 3 Fukushima Medical University, 1 Hikarigaoka, Fukushima-city, Fukushima Japan; 4 Department of Clinical Laboratory Medicine, Fukushima Medical University Hospital, 1 Hikarigaoka, Fukushima-city, Fukushima Japan

**Keywords:** Hospital-acquired infections, HAI, Operating rooms, ORs, Equipment contamination, Infections, Operating rooms, Bacterial infections, Anesthesia, Syringes

## Abstract

**Background:**

Some institutions reuse cuff syringes and do not periodically sterilize cuff pressure gauges. Pathogenic bacterial contamination of such equipment may increase the probability of pathogen transmission to patients during anesthetic procedures. Therefore, microbial contamination on cuff syringes, cuff pressure gauges, and their surroundings was assessed in the operating rooms of a university-affiliated tertiary care hospital in Japan.

**Methods:**

This study was conducted between April and May 2019 in 14 operating suites at a hospital. The following sites in each operating suite were sampled: cuff syringe (inner/outer components), outer components of cuff pressure gauge, cuff syringe and cuff pressure gauge storage drawers, and computer mice. The swabs were directly streaked onto agar plates and incubated. Then, the bacterial species were identified using mass spectrometry.

**Results:**

The highest bacterial isolation was observed in computer mice, followed by the outside of cuff pressure gauges and the drawers of cuff pressure gauges (92.9, 78.6, and 64.3%, respectively). Most of the identified bacteria belonged to the *Bacillus* species, with colonization rates of 85.7, 57.1, and 57.1% on computer mice, cuff pressure gauges, and cuff pressure gauge storage drawers, respectively. Coagulase-negative *Staphylococcus* was found in 35.7% of the specimens and was more prevalent on computer mice (71.4%), followed by on cuff pressure gauges (64.3%).

**Conclusion:**

Anesthesiologists should be aware of the possible pathogen contamination risk from cuff syringes, cuff pressure gauges, or associated equipment and take appropriate infection control measures to minimize the risk of pathogenic transmission.

## Background

Hospital-acquired infections (HAI) occur in 10% of inpatients [1] and increase postoperative morbidity and mortality [2, 3]. Pathogen transmission occurs via medical professionals, the hospital environment, and the equipment used by medical staff [4]. If the instruments or devices used in the operating room are contaminated, they can become sources of infection due to microbial transmission through disposables.

Loftus et al. reported that adjustable pressure-limiting valves and dials on anesthesia machines become severely contaminated by the time a procedure has concluded, thus possibly increasing the incidence of HAIs and mortality [5]. Additionally, the immunocompetence of patients under general anesthesia is temporarily diminished, rendering them more susceptible to infection [6, 7].

Some institutions frequently reuse syringes that inject air into intubation tube cuffs (cuff syringes) or often do not clean cuff pressure gauges or both. Thus, evaluating the contamination status of these instruments and routinely disinfecting them may be important for controlling HAIs. However, to the best of our knowledge, contamination of these devices has not been investigated thus far. Accordingly, we evaluated the bacterial contamination status on cuff syringes, cuff pressure gauges, and their surroundings in operating rooms.

## Methods

This study was conducted between April and May of 2019, at a 788-bed, university-affiliated hospital, which was a teaching hospital with 14 operating suites comprising 12 general operating rooms (ORs) and two bioclean rooms. The ORs handle various gastrointestinal, cardiovascular, neurological, and orthopedic surgeries, and 6,950 (5422 elective, 835 urgent, and 693 emergency) surgical procedures were conducted between 1 April 2018 and 31 May 2019. The average number of procedures conducted daily did not significantly differ between the ORs.

As the provision of anesthesia involves tasks such as attaching monitors to patients, intubating them, and placing infusion lines, anesthetists have to touch patients frequently. Hand hygiene is the most important and basic infection control measure. The World Health Organization recommends that hand hygiene be implemented at the following key time points: before touching patients, before disinfecting and starting aseptic procedures, after contact with body fluids, after touching patients, and after touching the surroundings of patients [8]. During the study period, the medical staff in the ORs took a course in hand hygiene based on The World Health Organization recommendations; the course is conducted twice annually. However, compliance with the hand hygiene rules was not officially evaluated. In addition, the staff wore a pair of disposable gloves at the provision of anesthesia; however, there was no rule regarding the time and frequency of changing the gloves.

**Fig. 1:**
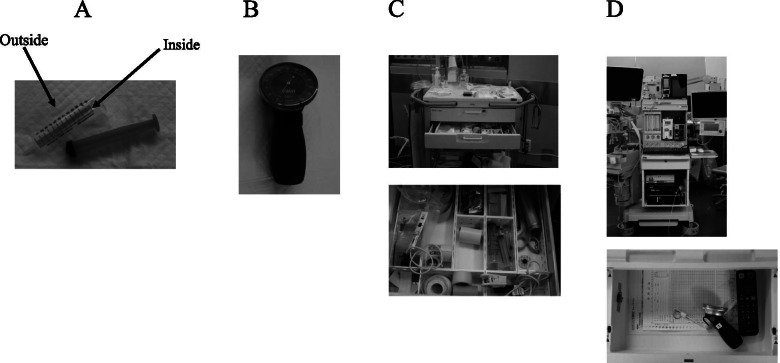
Equipment-sampling sites. **A** cuff syringe, **B** cuff pressure gauge, **C** drawer where the cuff syringes are stored, **D** drawer of the anesthesia machine where the cuff pressure gauges are stored

We assessed the following sites in the 14 ORs to determine the contamination status of cuff syringes, cuff pressure gauges, and their surroundings. Commercially available 10-mL syringes (Terumo syringe^TM^, Termo, Tokyo, Japan) were used as the cuff syringes in all of the ORs. The cuff syringes (Fig. 1a) were not single-use and were repeatedly reused in our hospital. After the operations were concluded, nurses disinfected the exterior of each instrument using sodium hypochlorite and replaced the instrument with a new one if any contamination was suspected upon visual inspection. However, no standard procedures had been established regarding the single or multiple usage of syringes, and the OR staff were not aware of the number of times a particular syringe had been used. Samples were obtained from the outer and inner components of the cuff syringes using Transwab® culture swabs (Medical Wire & Equipment, Corsham, UK) were cultured as described below.

The cuff pressure gauges (Endotrachael Tube Cuff Pressure Manometer, VBM Medical, Inc. Germany) (Fig. 1b) were not regularly disinfected, and visually appeared contaminated. The external surfaces of these cuff pressure gauges were swabbed and cultured as described below.

Nurses disinfected the drawers of the cabinets where the cuff syringes were stored (Fig. 1c) using sodium hypochlorite twice annually (August and December). Swab cultures were acquired from the compartments containing the cuff syringes.

The cuff pressure gauges were stored in drawers in anesthesia machines, and the cabinets had not been regularly disinfected. Swab cultures were acquired from the front right corners of the drawers.

Swab cultures were also acquired from the buttons of computer mice used to operate the electronic medical record system of each anesthesia machine (Fig. 1d). Anesthesiologists are required to frequently touch the anesthesia machine and electronic medical record system during anesthesia. The reason we examined contamination on computer mice is because it is absolutely necessary in the electronic anesthesia record system and is much more frequently used than a computer keyboard. The electronic medical record systems were not disinfected regularly.

One sample was obtained from each site listed above in each OR in the morning before commencement of surgery. The sampled materials were then streaked onto sheep blood agar (BD Columbia Agar with 5% sheep blood, Becton, Dickinson and Co., Franklin Lakes, NJ, USA) plates, followed by incubation of the plates at 35 °C for 24 h. Colonies were identified using a Microflex® mass spectrometer (Bruker, Billerica, MA, USA). The results were analyzed if the bacterial identification score was > 1.7.

As this study did not involve humans or animals, approval by an Ethics Committee was not applicable.

## Results

Overall, 60.7% of the samples were positive for bacteria; the rate was the highest for the computer mice followed by those for the outside of cuff pressure gauges and the drawers of cuff pressure gauges (92.9, 78.6, and 64.3%, respectively).

Most of the identified bacteria belonged to the genus *Bacillus*. The colonization rates of *Bacillus* were 85.7% in samples obtained from the computer mice, and 57.1% in those obtained from both the outer components of the cuff pressure gauges and the cuff pressure gauge storage drawers. Coagulase-negative *Staphylococcus* (CNS) was found in 35.7% of the samples, and the highest proportion of these organisms was found in the computer mice (71.4%), followed by the cuff pressure gauges (64.3%). Table 1 shows the culture results of each analyzed item.

**Table 1:** Identified bacterial sample aggregates

Room number	Organism(s) and biotype					
	Outer components of cuff syringes	Inner components of cuff syringes	Cuff pressure gauges	Drawers of cuff syringes	Drawers of cuff pressure gauges	Computer mice attached to anesthesiamachines
1	*-*	*-*	*Bacillus subtilis*	*Bacillus* spp.	*Bacillus* spp., *Paenibacillus cineris*	*Bacillus* spp., *Staphylococcus epidermidis*
2	*Staphylococcus caprae*	*-*	*-*	*-*	*-*	*Staphylococcus hominis*, *Staphylococcus epidermidis*
3	*-*	*-*	*Solibacillus silvestris*	*Bacillus* spp.	*Bacillus* spp., *Staphylococcus epidermidis*	*Bacillus* spp., *Staphylococcus epidermidis*
4	*-*	*-*	*Bacillus cereus*, *Bacillus subtilis*, *Staphylococcus capitis*	*-*	*Bacillus* spp., *Staphylococcus epidermidis*	*Bacillus* spp., *Staphylococcus* spp.
5	*-*	*-*	*-*	*-*	*Bacillus* spp.	*Bacillus* spp., *Staphylococcus epidermidis*, *Bacillus cohnii*
6	*-*	*-*	*Bacillus subtilis*	*-*	*-*	*Bacillus* spp., *Staphylococcus hominis*
7	*-*	*-*	*-*	*-*	*-*	*-*
8	*-*	*-*	*Bacillus subtilis*, *Staphylococcus epidermidis*	*Staphylococcus epidermidis*	*Bacillus* spp., *Bacillus flexus*	*Bacillus* spp., *Staphylococcus hominis*
9	*-*	*-*	*Bacillus subtilis*, *Staphylococcus epidermidis*	*-*	*Bacillus* spp., *Staphylococcus lugdunensis*, *Staphylococcus* spp.	*Bacillus* spp.
10	*-*	*Staphylococcus aureus*, *Staphylococcus capitis*, *Staphylococcus epidermidis*	*Staphylococcus epidermidis*	*Staphylococcus aureus*, *Staphylococcus caprae*, *Staphylococcus warneri*	*Bacillus* spp., *Staphylococcus epidermidis*	*Bacillus* spp.
11	*-*	*-*	*Bacillus subtilis*, *Staphylococcus capitis*, *Staphylococcus epidermidis*	*-*	*-*	*Bacillus* spp., *Staphylococcus epidermidis*
12	*Staphylocuccus capitis*	*-*	*Staphylococcus epidermidis*	*Bacillus halosaccharovorans*, *Staphylococcus epidermidis*	*Enterococcus faecalis*, *Staphylococcus aureus*	*Bacillus* spp., *Staphylococcus epidermidis*
13	*Staphylocuccus epidermidis*	*-*	*Bacillus cereus*, *Staphylococcus epidermidis*	*-*	*Bacillus* spp.	*Bacillus* spp.
14	*-*	*-*	*Bacillus subtilis*, *Staphylococcus hominis*	*Bacillus* spp.	*-*	*Bacillus* spp., *Staphylococcus capitis*, *Staphylococcus caprae*

Microorganisms were detected in 7.1% of the inner components (*n* = 1) and in 21.4% of the outer components (*n* = 3) of cuff syringes. *S. aureus*, *S. capitis*, and *S. epidermidis* were detected in the inner component of the cuff syringe used in OR number 10. We detected CNS including *S. caprae*, *S. capitis*, and *S. epidermidis* in the outer components of the cuff syringes used in OR numbers 2, 12, and 13.

We found that 78.6% of the cuff pressure gauges (*n* = 11) were externally contaminated (Table 1), and *Bacillus* spp. and CNS were detected from the cuff pressure gauges in almost all of the ORs. Bacteria were detected from 42.9% of the drawers where the cuff syringes were stored (*n* = 6), 64.3% of the drawers in which the cuff pressure gauges were stored (*n* = 9), and 92.9% of the computer mice (*n* = 13) (Table 1).

The bacteria detected from these locations included not only *Bacillus* spp. and CNS, but also various other bacteria including *S. aureus*, *S. lugdunensis*, and *Enterococcus faecalis*. In OR number 10, *S. aureus*, *S. caprae*, and *S. warneri* were detected on the drawers of cuff pressure gauges. Additionally, the bacterial diversity of colonies indicated high levels of contamination.

## Discussion

We evaluated the bacterial contamination status on cuff syringes, cuff pressure gauges, and their surroundings. The main bacterial species detected on the cuff syringes and cuff pressure gauges were skin bacteria such as *S. epidermidis*, *Bacillus* spp., and *S. aureus*, which were also detected in the surroundings of the cuff syringes and cuff pressure gauges.

*S. aureus*, *Bacillus cereus*, and the various CNS species detected in this study can all cause catheter-related infections. *S. aureus* is the main pathogen associated with HAIs. These pathogens can cause wound infections, infectious pericarditis, or respiratory tract infections in operating rooms [3]. *B. cereus* causes infections including endophthalmitis and sepsis [9]. The most concerning pathogen among the CNS bacteria is *S. lugdunensis*, because like *S. aureus*, it adheres to devices such as catheters and artificial substances, and causes serious infections including sepsis, and skin and soft tissue infections [10]. Although *S. epidermidis* is comparatively a less concerning pathogen, it may cause device-related infections [11]. The intestinal microorganism *E. faecalis* is rarely detected on environmental surfaces under normal circumstances, and its detection suggests environmental contamination caused by the hands of the medical staffs.

Although the outer components of cuff syringes were supposed to be disinfected at the end of each surgical procedure, it is noteworthy that some colonization was detected on the outer components. No rules stated that the inner components of cuff syringes must be cleaned because the inner components of cuff syringes were thought to rarely contact the outside and a difficult shape to clean up. However, the finding of *S. aureus* as well as other CNS species on the inner components of cuff syringe is also noteworthy. This identified contamination was probably caused by repeated use of the same syringe for long periods without disinfecting its interior. In fact, the drawer used for storing cuff syringes in OR 10 was severely contaminated with bacterial species including *S. aureus*. Moreover, it is reasonable to think that contaminations were detected much more frequently on the other devices investigated in this study and their surroundings because they had been rarely disinfected before.

As most of the bacteria detected in this study could cause infections in patients with diminished immunocompetence who are undergoing general anesthesia in the OR, improvements are required to ensure a safe anesthetic environment. In light of these findings, the following improvements should be considered. Firstly, these presented findings should be shared during a meeting with all anesthetists working at our hospital. In addition, the staff should be instructed to take much greater care with regard to hand hygiene. Next, since syringes are inexpensive (~USD $0.40 each) using them only once and discarding them appropriately is highly recommended. Our hospital has adopted the “single-use only” rule for cuff syringes. Moreover, sodium hypochlorite exerts a disinfectant action through the oxidative decomposition of bacteria and viruses, and it is effective even against sporulating bacteria such as *B. cereus*. Drawers of cabinets containing cuff syringes, cuff pressure gauges, drawers where they were stored, and computer mice must be regularly disinfected with sodium hypochlorite.

As another option, environmental contamination can reportedly be reduced by wearing two pairs of gloves to induce general anesthesia and removing the outer set after contact with the patient and before using the anesthesia machine [12]. Most of the identified bacteria were known skin commensals. Therefore, the double glove technique might reduce the contamination of the equipment used during anesthesia.

It is likely that in many hospitals, cuff syringes are changed for each patient, and pressure gauges are cleaned regularly. However, it is true that a few hospitals still do not change and clean these items, and there are no clear, relevant guidelines. Therefore, it is important to emphasize that we should do while presenting persuasive data. Our results might be predictable because every equipment that is used repeatedly has possible pathogen contamination risk. However, our data would be valuable due to a limited number of reports showing that pathogen contamination on anesthesia-associated equipment certainly exists. Therefore, it is important to recognize that pathogen contamination arises in anesthesia-related surroundings. Regarding sterilizing or replacing anesthesia-associated equipment, each hospital must have a standardized procedure that is suitable to the hospital conditions and circumstances.

This study has at least three limitations. First, the bacterial counts could not be accurately measured. Second, only one sample was obtained from each site in the 12 ORs. Third, we could not establish a relationship between equipment contamination and the occurrence of patient infections.

## Conclusions

We evaluated bacterial contamination on peripheral equipment used for anesthesia in the OR at our hospital. This equipment was not regularly replaced or cleaned at specific times. We identified contamination with *S. aureus*, *B. cereus, S. lugdunensis*, *E. faecalis*, and *S. epidermidis* in our samples. Based on our results, we recommend that cuff syringes should be replaced for every patient and cuff pressure gauges should be cleaned regularly.

## Data Availability

All data generated or analyzed during this study are included in this published article and its supplementary information files.
